# A Case of Tizanidine Withdrawal Syndrome: Features and Management in the Emergency Department

**DOI:** 10.7759/cureus.49248

**Published:** 2023-11-22

**Authors:** Marwa Morgom, Doaa M Sabir, Hanna Elbashir, Leena Saeed, Amal Alamin, Yara Abuazab, Nadir Abdelrahman

**Affiliations:** 1 Emergency Medicine, Hamad General Hospital, Doha, QAT; 2 Internal Medicine, Hamad Medical Corporation, Doha, QAT; 3 Family Medicine, Michigan State University College of Human Medicine, East Lansing, USA; 4 Medicine and Surgery, Jordan University of Science and Technology, Irbid, JOR; 5 Family Medicine, Hamad General Hospital, Doha, QAT; 6 Family Medicine - Geriatrics, Michigan State University College of Human Medicine, East Lansing, USA

**Keywords:** withdrawal syndrome, alpha 2-adrenoceptor agonists, adrenergic effect, tizanidine withdrawal, tizanidine

## Abstract

Anxiety medications, muscle relaxants, and sleeping pills have the potential to cause complications, side effects, and withdrawal symptoms if not prescribed and managed appropriately. Tizanidine, a short-acting muscle relaxant, acts on central alpha-2-adrenergic receptors to reduce spasticity. However, abrupt withdrawal of tizanidine can lead to symptoms such as hypertension, reflex tachycardia, hypertonicity, and anxiety as a result of high adrenergic activity. Few cases have been reported on tizanidine withdrawal syndrome. Here, we are presenting a rare occurrence of tizanidine withdrawal syndrome in a patient presenting to the emergency department with vomiting, generalized tremor, dysthermia, hypertension, and tachycardia. We discuss the management approach used to stabilize the patient and successfully control the symptoms by reintroducing a low therapeutic dose of tizanidine.

## Introduction

Tizanidine is an imidazole derivative with central analgesic action used as a muscle relaxant to treat muscle spasms and chronic spasticity. It has a similar structure to clonidine and strongly binds to α2-agonist and imidazoline (I) receptors [1]. Presynaptic inhibition, by reducing the nervous reflex, has the ability to act as an analgesic [2]. Tizanidine's muscle relaxant effects, which are evident in its suppression of spinal reflexes, are mediated by imidazoline receptors [3].

Tizanidine withdrawal results in a rebound peak in circulating catecholamine levels in the blood, which causes hypertension, tachycardia, and Increased spasticity [4]. However, tizanidine withdrawal syndrome is uncommon, with only a few cases reported in the literature. Sudden discontinuation of tizanidine increases the risk of developing withdrawal syndrome. Therefore, it is advisable to taper off the medication rather than abrupt cessation. 

We report a case of a 29-year-old male who presented to the emergency department with symptoms of adrenergic overstimulation, which was found to be due to tizanidine withdrawal.

## Case presentation

This case involves a 29-year-old male patient without any history of drug abuse or chronic disease. He did have a history of insomnia. He had sought treatment from a psychiatrist and was prescribed tizanidine low dose for insomnia. Unfortunately, due to poor follow-up, he continued taking this medication at a significantly higher dose (300 mg daily) than the recommended daily limit of 36 mg. The patient was on tizanidine for seven months. The patient presented to the emergency department with symptoms including vomiting, continuous hiccups, and fever. These symptoms began approximately 10 hours after he missed his last dose of tizanidine, which was unavailable to him at the time.

On clinical evaluation, the patient was in acute distress, presenting with a fever of 38°C, blood pressure of 200/150, and a pulse rate of 160 beats per minute. The patient also had a respiratory rate of 25 breaths per minute and maintained saturation on room air. Physical examination revealed an alert and oriented patient with a Glasgow Coma Scale score of 15/15. However, the patient appeared agitated with a flushed face and sweaty palms.

**Figure 1:**
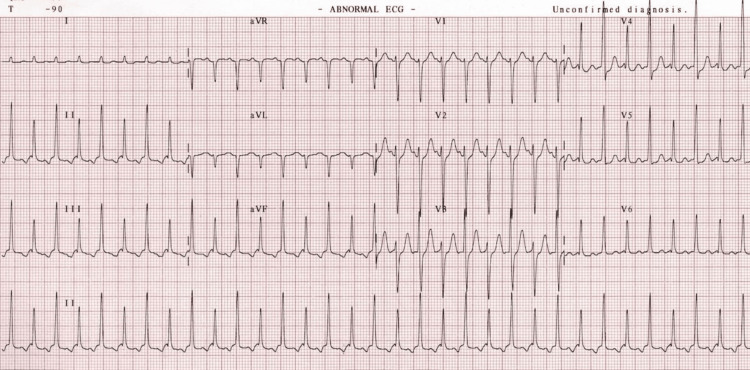
ECG done at presentation showing supraventricular tachycardia.

**Figure 2:**
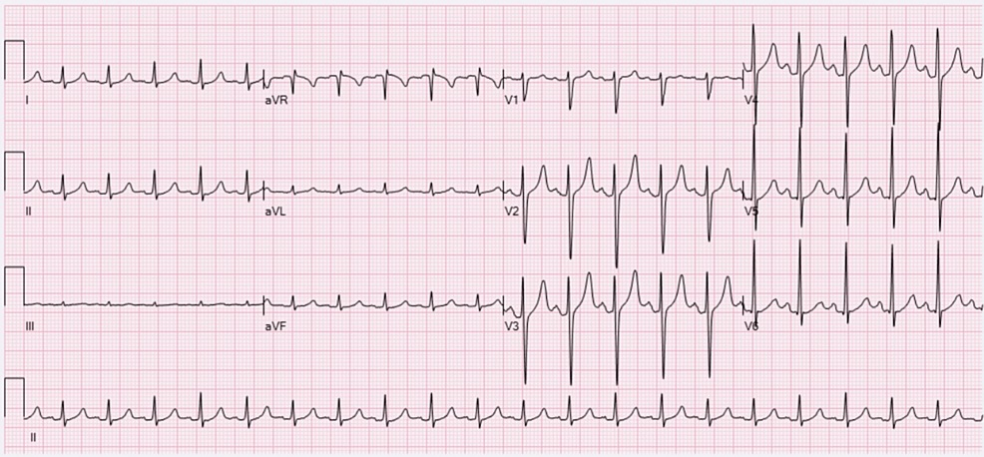
Repeated ECG showed sinus tachycardia.

Initial investigations showed supraventricular tachycardia (SVT) in the electrocardiogram (ECG) (Figure 1), which later reverted to sinus tachycardia (Figure 2). Routine laboratory tests were conducted, including complete blood count (CBC), renal function test, serum electrolytes, and serum creatine phosphokinase (CPK) level. These tests indicated elements of dehydration along with mild liver and kidney injury (Table 1). Ethanol levels were within normal limits, and toxicology screening yielded negative results.

**Table 1:** Relevant laboratory tests.

Laboratory Test	Before Treatment	After Treatment	Reference Range
Creatinine	90	80	62-106 umol/L
Blood Urea Nitrogen (BUN)	8	3	2.5-7.8 mmol/L
Potassium	5	4.5	3.5-5.3 mmol/L
Sodium	148	140	133-146 mmol/L
Creatinine Kinase (CK)	2000	200	39-308 U/L
Myoglobin	80	26	28-72 ng/ml
Aspartate Aminotransferase (AST)	80	30	5-34 U/L
Alanine Aminotransferase (ALT)	60	20	0-55 U/L
Alkaline Phosphatase (ALP)	150	120	40-150 U/L
Total Bilirubin	4	1	0-21 umol/L
Lactic Acid	6.7	3.20	0.5-2.2 mmol/L

The management plan started by controlling the patient’s symptoms and supportive measures started first by using cold sponging and intravenous (IV) paracetamol for hyperthermia, as well as administration of 2 mg IV lorazepam to control agitation and restlessness. The patient received a total of 6 mg IV lorazepam during his stay in the resuscitation area. Labetalol 10 mg was administered as needed to manage his elevated blood pressure. The second step after stabilization was to switch all the IV medications to the oral route of administration, including lorazepam 4 mg before sleep, metoprolol 75 mg twice daily, mirtazapine 15 mg once daily, and metoclopramide 10 mg as needed. Lastly, tizanidine was reintroduced at a dose of 4 mg three times daily, with a planned tapering of 2 mg per day.

Patient symptoms resulted from sudden adrenergic discharge triggered by tizanidine withdrawal syndrome. The decision to reintroduce tizanidine with lower doses and gradually tapering down led to significant improvement in the patient's restlessness and vital signs. The patient was shifted to the medical ward in stable condition after six hours in the high acuity unit in the ED, and was discharged the next day on amlodipine 5 mg and clonidine 0.15 mg three times a day, with an urgent psychiatry appointment scheduled for follow-up.

## Discussion

Tizanidine is considered an α2 receptor agonist that inhibits noradrenaline release. It’s an FDA-approved drug to treat chronic spasticity and muscle spasms caused by multiple sclerosis, a spinal cord injury, or an acquired brain injury. It has an analgesic effect which is used in managing chronic neck and lumbosacral neuralgia. It is also prescribed off-label for insomnia and migraine headaches and as an anticonvulsant. The side effects of tizanidine include dry mouth, dizziness, elevated hepatic transaminases, bradycardia, hypotension, hallucination, and sedation [4-8].

Tizanidine withdrawal syndrome occurs as a result of an adrenergic surge that is due to inhibition of the chronic blockade of adrenaline release. This adrenergic surge causes hemodynamic instability that manifests as refractory hypertension, tachycardia, and severe spasticity. Hence, it is advisable to avoid abrupt withdrawal of tizanidine therapy, particularly in patients treated with higher doses as they are more prone to develop the withdrawal syndrome [7]. This was the case with our patient, who was taking a high dose of tizanidine, and this made him more vulnerable to developing withdrawal symptoms. 

Management of tizanidine withdrawal syndrome includes two main components [9]. The first component is the hemodynamic control with adrenergic blocker drugs, and it is advisable to combine α-blockers and β-blockers to avoid aggravating hypertension [10]. The second component is to reintroduction of tizanidine in a lower dose followed by a gradual dose titration [4].

Tizanidine withdrawal is not a very common condition. However, it may have serious outcomes. Our literature review showed a few reported cases with a similar presentation to our case [4,7,8]. Early recognition of symptoms as well as prompt treatment are the mainstay for patient survival [4-8].

## Conclusions

This report describes the case of a 29-year-old male who presented with symptoms of tizanidine withdrawal syndrome, which is a very rare but serious presentation. The case report highlights the importance of recognizing tizanidine withdrawal symptoms. In addition, the importance of close monitoring, hemodynamic control, and reintroduction of tizanidine drug at a lower dose followed by tapering down for managing tizanidine withdrawal is emphasized. It is essential to consider the possibility of withdrawal syndrome when abruptly stopping any CNS-involved medications.
